# Complete Nucleotide Sequence of a French Isolate of *Maize rough dwarf virus*, a *Fijivirus* Member in the Family *Reoviridae*

**DOI:** 10.1128/genomeA.00623-16

**Published:** 2016-07-21

**Authors:** L. Svanella-Dumas, A. Marais, C. Faure, S. Theil, J. B. Thibord, T. Candresse

**Affiliations:** a UMR 1332 Biologie du Fruit et Pathologie, INRA, Univ Bordeaux, Villenave d’Ornon, France; b ARVALIS, Institut du Végétal, AGROSITE de Pau-Montardon, Chemin de Pau, Montardon, France

## Abstract

The complete nucleotide sequence of a French isolate of *Maize rough dwarf virus* (MRDV) was determined by next-generation sequencing and compared with the single available complete sequence and with the partial sequences of two additional isolates available in online databases.

## GENOME ANNOUNCEMENT

*Maize rough dwarf virus* (MRDV), a *Fijivirus* member in the family Reoviridae (1), was first reported from the south of France and Corsica in the late 1970s (2) and occurs in Europe and the Middle East (3). Infected corn (*Zea mays* L.) is severely dwarfed and stunted with malformed leaves, poor root development, and important yield reduction.

Here, the sequences of the 10 genomic segments of a French MRDV isolate are reported, representing the second complete genome sequence for this poorly characterized agent. Double-stranded RNAs from symptomatic maize leaves collected in 2015 were purified, randomly amplified, and sequenced using the Illumina platform, according to Candresse et al. (4). After quality trimming, the reads were assembled into contigs using the CLC Genomics Workbench 7.0 and in-house-developed pipelines. The contigs were annotated by a BlastX and BlastN comparison with GenBank. A single large scaffold was reconstructed for each genomic segment, integrating data from a total of 53,694 reads (71.1% of the total reads). For a few segments, the 5′ and 3′ ends were determined using a Rapid Amplification of cDNA Ends (RACE) strategy, according to the kit manufacturer’s instructions (TaKaRa Bio Europe/Clontech, France).

The genome of MRDV, coding for 13 viral proteins, is composed of 29,145 nucleotides (nt), 1 nt longer than the recently reported complete genomic sequence of an Italian MRDV isolate collected in 2008 (accession numbers HQ637550 to HQ637559) (5). For each segment, the conserved extreme 5′- (5′-AAGTTTTTT) and 3′- (CAGCT[A/G][A/T][T/C]GTC-3′) end sequences were retrieved and are similar to those reported, not only for MRDV isolates, but also for the other group 2 fijiviruses (6). At the nucleotide sequence level, the 10 genome segments of the French isolate show identity levels ranging from 94.5% to 99.2% with the corresponding segments of the Italian MRDV isolate, and from 95.4% to 98.8% with the partial sequences for segments S1 to S9 and the full sequence of segment S10 of the Spanish MRDV isolate LSP collected in 1999 (accession numbers LK392325 and LK392331 to LK392343) (7). Furthermore, a comparison performed on the segment S10 sequences from six additional Spanish MRDV isolates collected from 2005 to 2010 (accession numbers JQ975001 and LK392326 to LK392330) (7) shows a within-group nucleotide identity ranging from 97.6% to 99.9%, which is in the same range of the nucleotide identity found between French isolate and MRDV LSP S10 sequences (98%). Focusing on the encoded proteins, all but three (P4, P7-2, and P8) show a very high level of amino acid identity (>98.5%) between the French and Italian MRDV isolates. Remarkably, P4 and P7-2, which show only 97.6% and 93% amino acid (aa) identity, respectively, with the Italian MRDV sequences, display 99.4% and 99.6% aa identity, respectively, with the proteins of the LSP Spanish isolate, which might indicate the existence of pseudorecombination events between MRDV isolates. In conclusion, the European MRDV isolates collected over a period of 10 years in three different countries appear to show a high level of homology, suggesting a low temporal and geographical variability of this poorly characterized virus.

### Nucleotide sequence accession numbers.

The complete genomic sequence of MRDV reported here has been deposited in GenBank under accession numbers KU984966 to KU984975, corresponding to genomics segments 1 to 10, respectively.
